# Nail Scissor as a Rare Foreign Body in the Urethra: Case Report

**DOI:** 10.7759/cureus.3851

**Published:** 2019-01-08

**Authors:** Betul Cam, Hakan Tuncer, Ozlem Uzun, Emin Uysal

**Affiliations:** 1 Emergency Medicine, Bagcilar Education and Research Hospital, Istanbul, TUR

**Keywords:** foreign body, self-applied nail scissor., urethra

## Abstract

Foreign bodies in the urethra are rare in the literature. A majority of the foreign bodies administered in the urethra are because of a psychiatric disorder, senility, intoxication, and self-erotic stimulation. Clinical examination and imaging tests, such as X-ray, ultrasound, computed tomography (CT), and magnetic resonance imaging (MRI) have been used for the diagnosis of foreign bodies. Surgical exploration or endoscopic extraction are the main approaches to the treatment. This case report deals with a 45-year-old male patient who was admitted with urethral pain to the emergency service. A nail scissor was diagnosed in the urethra and endoscopic extraction was performed under regional anesthesia.

## Introduction

Foreign bodies can be diagnosed incidentally or while seeking the origin of discomfort related to an inserted body [1]. A foreign body in the lower urinary tract has been reported rarely in the literature [2]. Several cases of the insertion of foreign bodies, such as pins, electrical wires, screws, seeds of olives, or ballpoint pens, have been reported previously [3-7]. Imaging tests help clinicians diagnose many foreign objects in the human body [8-9]. Most patients come to the hospital with diverse complaints, such as hematuria, dysuria, urinary frequency, strangury, and urinary retention [1-7]. The majority of foreign bodies in the lower urinary tract have been administered because of a psychiatric disorder, senility, intoxication, and self-erotic stimulation [2]. Patients have been found commonly in the adult male population. We report a single case of a 45-year old male patient who inserted a nail scissor in his urethra. He was admitted to hospital with urethral discomfort and pain.

## Case presentation

**Figure 1:**
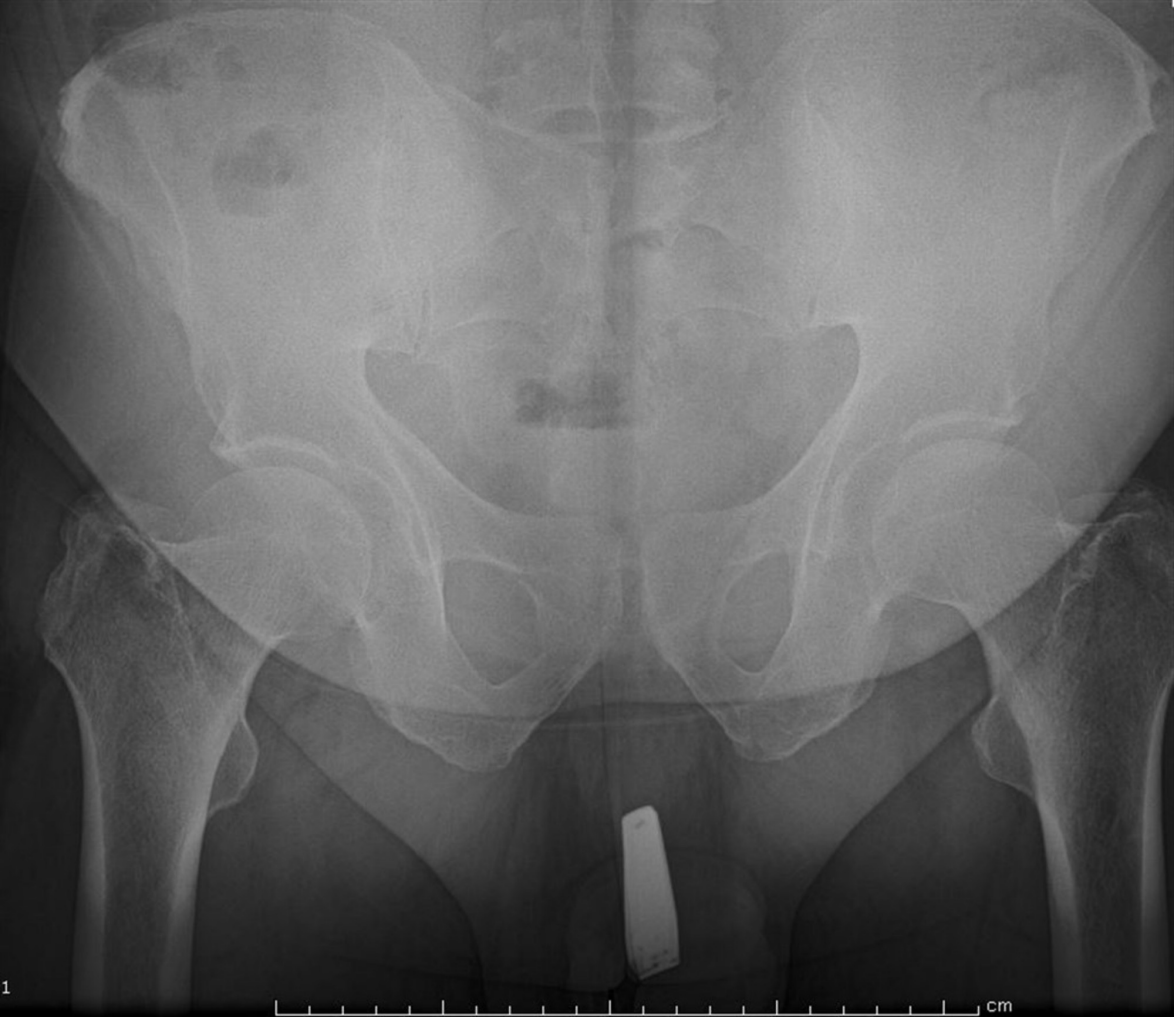
Radiological imaging of foreign body in the urethra.

**Figure 2:**
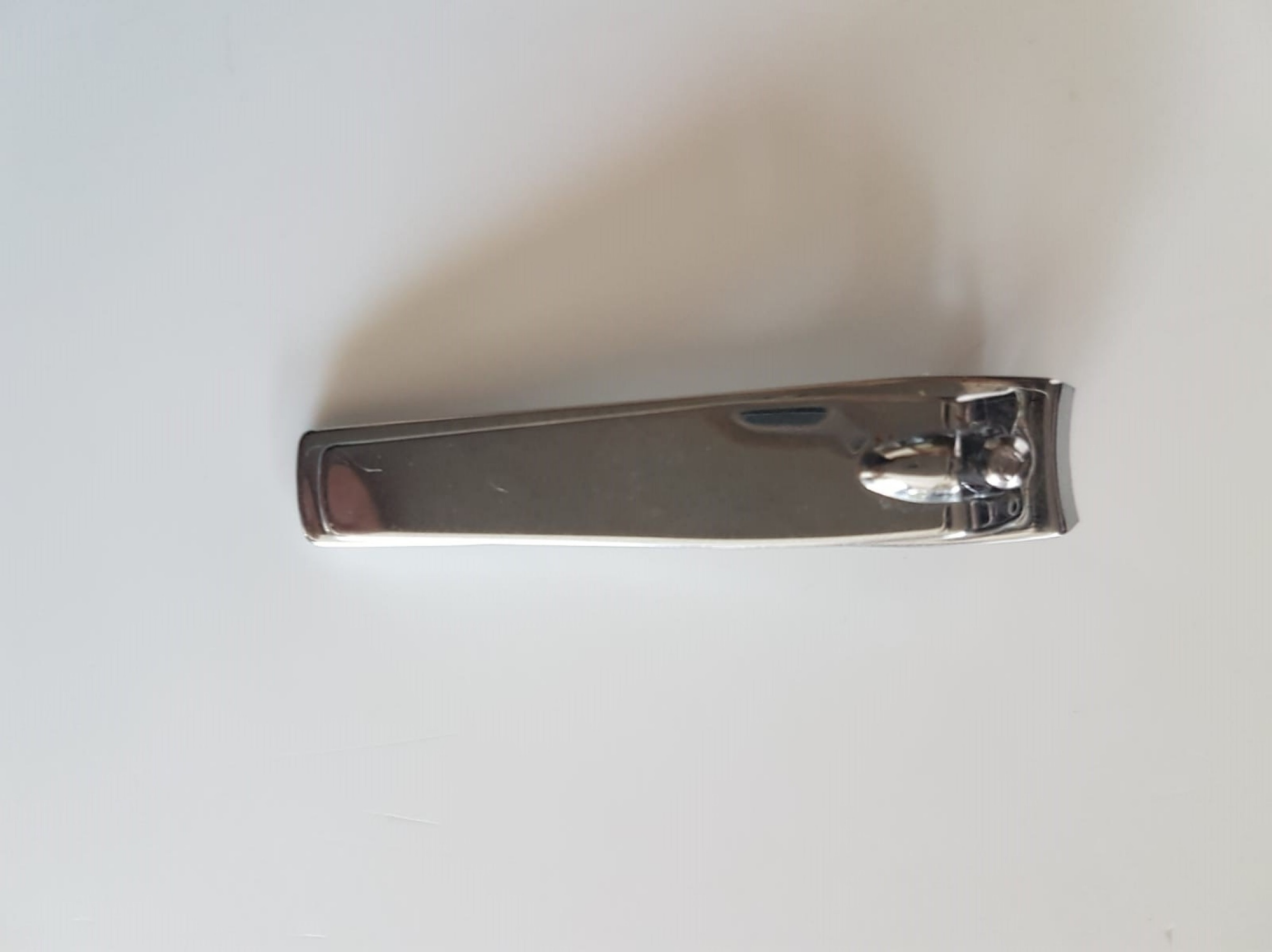
Nail scissor removed from the urethra.

A 45-year-old male patient applied to the emergency service with a nail scissor inserted in the urethra. The patient gave a history of self-insertion of the instrument in the urethra. There were urethral bleeding and pain with the normal passage of urine. A hard-edged foreign body was pulped by urethral examination. A radiographic image was taken (Figure 1) and a nail scissor was diagnosed in the urethra. Urethral extraction was applied under local anesthesia to remove the foreign body (Figure 2). An image could not be taken during the operation because the patient disapproved. Antibiotherapy and analgesia were given to the patient following surgery. The next appointment was set to check for urethral healing in the urology department.

## Discussion

Many different foreign bodies have been diagnosed in the male urethra. Self-inserted wires, screws, nuts, a metallic tongue cleaner, and pens have been reported previously [1-7]. Imaging tests, such as X-ray, ultrasound, CT, and MRI, are used to determine the bodies in the genitourinary tract [8-9]. Ultrasound could be preferred since it is a cheaper, readily available, and non-invasive technique in the emergency department [8]. The endoscopic and surgical interventions are used for the removal of foreign bodies [10-13]. An endoscopic procedure under local anesthesia is a fast and recommended method for removing foreign bodies, such as wires, pins, screws, and pens, from the urethra. Surgical intervention may also be required in some cases for irregularly shaped large bodies, such as a metallic tongue cleaner, pens, or keys. The following is the extraction antibiotic treatment needed. Psychiatric disorders should be sought in cases of habitual recurrence [14]. A nail scissor is a very rare case in the urethra (Figure 1). The forcing of a nail scissor into the urethra could be harmful due to its hard edges and sharpened side. In this case, a sharp-sided object was removed under local anesthesia, as shown in Figure 2.

## Conclusions

In conclusion, self-applied urethral foreign body insertion is a rare case. Imaging tests are needed for determining the urethral location, shape, and size of the bodies. Urethral stricture might be taken into account after any surgical intervention despite the removal of the foreign body with little damage. Finally, psychiatric counseling should be recommended for these patients.
